# A Comparative Bryo-Ecological Study of Habitat 3170*: Sites of Particular Phytogeographic Interest in the Mediterranean Area

**DOI:** 10.3390/plants13152113

**Published:** 2024-07-30

**Authors:** Silvia Poponessi, Daniela Gigante, Annalena Cogoni

**Affiliations:** 1 Department of Environmental and Life Science, Botany Section, University of Cagliari, Viale S. Ignazio, 13, 09123 Cagliari, Italy; 2 Eurac Research, Institute for Alpine Environment, 39100 Bolzano, Italy; 3 Department of Agricultural, Environmental, and Food Science, University of Perugia, Borgo XX Giugno, 74, 06121 Perugia, Italy; daniela.gigante@unipg.it

**Keywords:** bryophytes, climate changes, ephemeral liverworts, functional characteristics, habitat monitoring, Mediterranean temporary ponds

## Abstract

In accordance with the 92/43/EEC “Habitats” Directive, Mediterranean temporary ponds are identified as a priority natural habitat within the European context. They are a very interesting and unique habitat type, as ecological conditions can vary greatly in a short period of time. Due to their small size, many Mediterranean hydrophytic bryophytes typical of this habitat are often overlooked or misinterpreted. Their distribution, habitats, ecology, and strategies are generally poorly understood. Several of them are currently considered rare or endangered in the Mediterranean. As these ponds are particularly sensitive to human activities and natural changes, such bryophytes and associated vegetation communities may be at risk. This study is focused on their floristic variability in different environmental conditions in two sites of particular phytogeographic interest in the Mediterranean area. In the Sardinian *Pauli* of Giara, 56 taxa (50 Bryophyta and 6 Marchantiophyta) were found, and in the Umbria Piana di Ferretto, 54 taxa (34 Bryophyta and 20 Marchantiophyta) were documented. The taxa from the two areas were analysed and compared. Life strategies, life macroforms, light and moisture preferences, chorological elements, and moisture belts were considered. The data are presented here together with information on the phytogeography and ecology of the species recorded. The findings indicate that a bespoke monitoring strategy and dedicated conservation measures are essential for the effective protection of bryophytes, ensuring the achievement of meaningful and sustainable conservation outcomes.

## 1. Introduction

**Figure 1:**
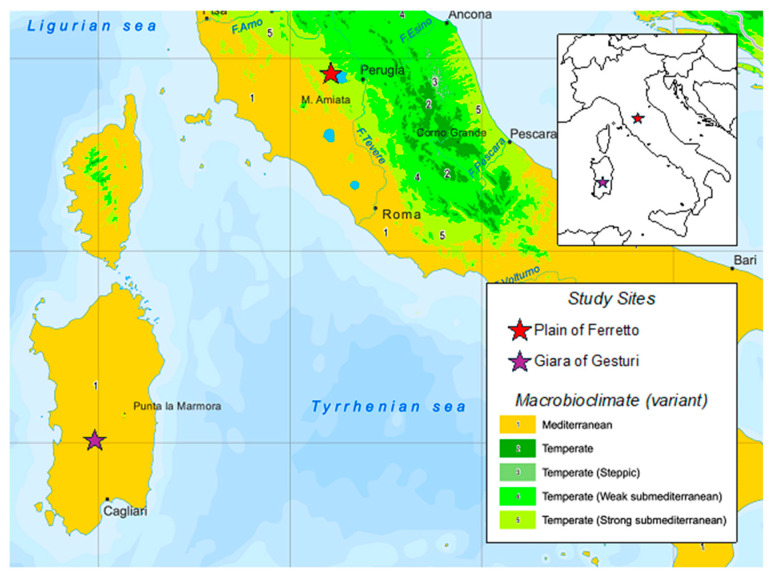
Study areas and Macrobioclimate variant [13].

Recently, an increasing amount of research is being conducted on plant communities growing in temporary ponds and their associated adaptations to the adverse conditions during the dry phase. This provides an excellent opportunity to examine the effects of habitat duration and fluctuating water levels on community structure and bryophyte species living in this particular habitat. Mediterranean temporary ponds (MTPs) are functionally distinct from all other wetlands and can contribute significantly to regional biodiversity, supporting a relevant number of species compared to other types of water bodies [1]. They are fragile environments, highly dependent on water fluctuations and are often localised in small areas [2]. Although there has been a recent increase in awareness of the importance of these environments, appropriate protection measures are only sometimes taken [3,4,5]. MTPs are of major conservation importance, because, despite their small size, they shelter many rare and endangered species. Another important reason supporting the necessity of their protection is that these ponds show an alarming rate of disappearance and degradation [6]. Due to their small size and simple community structure, temporary pools are often considered as early warning systems of the effects of long-term changes to larger aquatic systems [7,8]. According to the European Habitat Assessment, at the biogeographical level [9], the overall evaluation of Habitat 3170* in Italy is “Unfavourable-Bad” in both the Mediterranean and Continental biogeographical regions. Italy represents a critical area for this habitat, considering its central position in the Mediterranean basin. In the European Red List of Terrestrial Habitats [10], based on the available information, the habitat was assessed as VU based on criteria C/D1 for EU28 and EU28+. The critical status of this habitat type is a consequence of its intrinsic characteristics. It occurs in a relatively small area across a wide range with a highly fragmented distribution. It is often poorly identified, and its importance is largely unappreciated, leaving it vulnerable to unintentional destruction [11]. The majority of temporary ponds are shallow, rendering them susceptible to destruction. Their biological balance can be suddenly altered by many factors that occur both gradually and also suddenly in these areas [4]. Their small water volumes influence their high susceptibility to pollution but also to climate changes, which can influence these biocenoses in the medium–long term. Among the major pressures and threats, the natural vegetation processes that lead to an expansion of the shrubs have also been recognized, as well as the accumulation of litter on the ponds’ bottom. Additionally, a serious threat recently recognised is the presence of alien species, such as the moss *Campylopus introflexus* (Hedw.) Brid., which forms compact felts on the soil, dominating the characteristic and ephemeral species of the pools [12]. Among the disturbing factors affecting this habitat, waste abandonment should also be included, together with all those practices that interfere with the naturalness of a territory. The purpose of the article is to better understand the biology and ecology of these very fragile ecosystems (structure and function and typical species), which are at present only partially known, to inform and address possible management solutions and intervention techniques that are respectful of the delicate balances that allow for the maintenance of a good conservation status. In this frame, we tried to understand how the habitat’s floristic and structural characteristics differ in the various environmental contexts and especially in different macroclimates, with a special focus on the bryophytic component Figure 1.

## 2. Results

**Figure 2:**
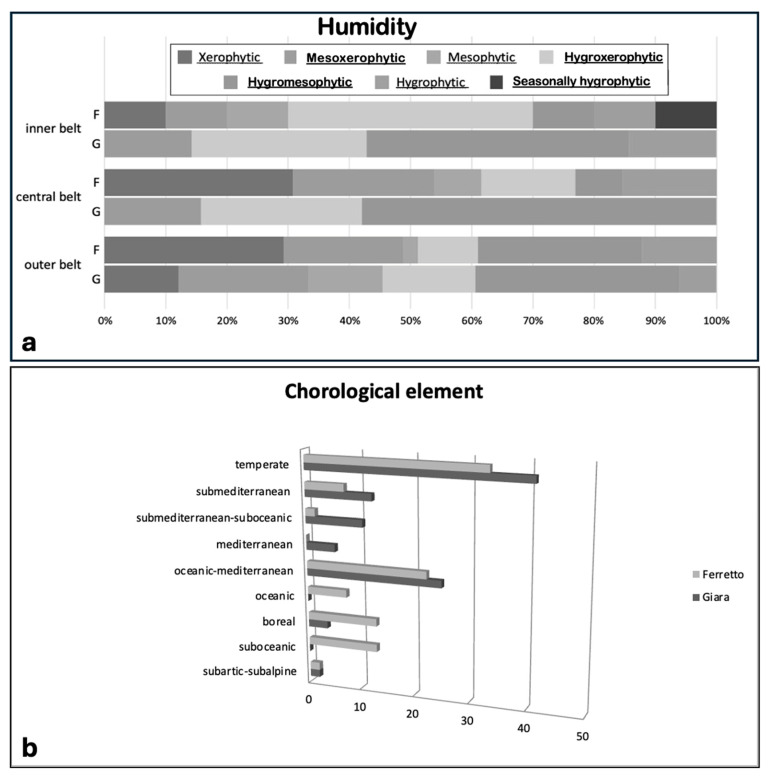
(**a**) Humidity per belt to Giara and Ferretto by [11]; (**b**) Chorological element based on [18].

The study of the bryophytes in MTP systems has highlighted the presence of species that are mostly diversified according to their water requirements. As a consequence of the rapid life cycle and seasonal water availability, many bryophytes growing in this habitat are adapted to survive complete submersion, being exposed to the atmosphere for several months, and periods of desiccation. This often requires physiological and morphological adaptations for survival in a range of different microhabitats. These can include free floating in water, growth on the surface of submerged objects, and growth on substrates from temporarily emergent to completely submerged. These species are ephemeral, inhabiting depressions in the substrate and exhibiting sensitivity to fluctuations in precipitation patterns. The species’ dependence on the specific substrate type and the duration of flooding is well documented. However, the extent of their tolerance to anthropogenic disturbances remains variable. A synthesis of the results obtained is shown in Table 1. The total number of species recorded in the two sites is 97, distributed in 54 units in Ferretto and 56 in Giara. All the species found during the surveys in the two sites are reported in the table in alphabetical order. For each species, we added the ecological requirements and the biological attributes. The last columns represent the occurrence of the species in the sites. The ecological characteristics of the species are highlighted, and ecological data are compared between sites. In the two sites, the number of species exclusive of one belt varies significantly from the inside to the outside of the pool. Additionally, the number of species per belt in the two sites is not equal. At Giara, in the outer belt, we found the highest number of species (42). As we move inland, we went from 13 to 10 species (central and inner belts, respectively). At Ferretto, the outer belt comprises 34 species, the central belt 19, and the inner belt 7. In the inner belt of Ferretto, there are only two exclusive species, while in Giara, there are eight8 species. In the central belt of Ferretto, we found 11 exclusive species, while in that of Giara, there are 6. As far as the outer belt is concerned, it is the most species-rich of both sites. At Ferretto, we found 29 exclusive species, and at Giara 34; these observations are shown in Figure 2a,b. We observed how the internal belt in both sites is the one with exclusive species: here we did not find species common to the two sites. The intermediate belt sees only two taxa in common: *Tortula truncata* and *Archidium alternifolium*. *Hypnum cupressiforme*, *Funaria hygrometrica*, *Barbula unguiculata*, *Tortula muralis*, *Didymodon luridus*, *Tortella squarrosa*, *Riccia sorocarpa*, *Imbribryum alpinum,* and again *Archidium alternifolium* are the species in common between the external belts of the pools of the two sites.

The categorisation of bryophytes according to their capacity to occupy, reproduce, and persist in a specific environment is accomplished through the utilisation of life strategies.

**Figure 3:**
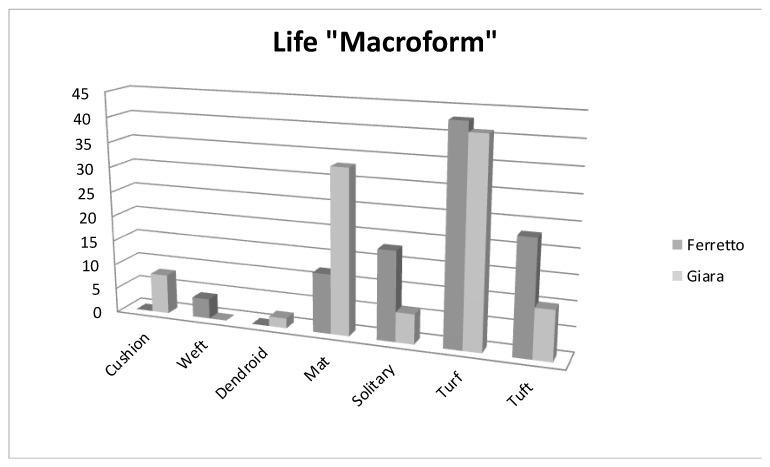
Life macroforms adapted to the bioclimatic situation of the two sites.

Given that MTPs are ephemeral habitats, life strategies inherently entail a series of trade-offs, manifesting in a range of traits, including the capacity to either avoid or tolerate stressful environments, the preference for sexual or asexual reproduction, the length of the lifespan, and the size of the spore [14]. Along a gradient from avoidance to tolerance, the life strategies adopted by bryophytes determine different responses to environmental disturbance. In Giara, 83% of the species are perennial, against 64% in Ferretto; this, compared with the data on the life macroforms, indicates that the species characteristics of these environments are strictly ephemeral but remain in the soil in the form of spores and then germinate in favourable conditions. In fact, the most characteristic species are represented by a *turf* strategy. Life macroforms were merged [15] and adapted to the bioclimatic situation of the two sites (Figure 3).

**Table 1:** Taxa present at Giara and Ferretto; Humidity needs, Life cycle (A = annual, AP = annual or perennial, P = perennial, and PA = perennial or annual), and Life form [Solitary creeping (Sc), Solitary thalloid (St), Turf protonemal (Tp), Turf scattered (Ts), Turf vertical stem (Tf), Tuft (Tuft), Cushion (Cu), Dendroid (De), Mat rough (Mr), Mat smooth (MS), Mat thalloid (Mt), and Weft (We)] according to the classification in [15]. Life strategy follows [16]. Chorological elements follow [17].

Taxa	Humidity	Chorological Element	Life Cycle	Life Strategy	Life Form	Giara	Ferretto
*Anomodon viticulosus* (Hedw.) Hook. & Taylor	Meso-xerophytic	Suboceanic	P	p	Mr		x
*Archidium alternifolium* (Hedw.) Mitt.	Hygro-xerophytic	Suboceanic	PA	s	Tf	x	x
*Atrichum undulatum* (Hedw.) P.Beauv.	Hygro-mesophytic	Temperate	P	s	Tf		x
*Barbula unguiculata* Hedw.	Hygro-xerophytic	Temperate	P	c	Tf	x	x
*Bartramia pomiformis* Hedw.	Hygro-mesophytic	Boreal	P	l	Tuft	x	
*Bartramia aprica* Müll.Hal.	Xerophytic	Oceanic-mediterranean	P	l	Tuft	x	
*Brachytheciastrum velutinum* (Hedw.) Ignatov & Huttunen	Hygro-mesophytic	Temperate	P	cp	Mr	x	
*Brachythecium rutabulum* (Hedw.) Schimp. var. *rutabulum*	Meso-xerophytic	Temperate	P	p	Mr	x	
*Bryum dichotomum* Hedw.	Meso-xerophytic	Submediterranean	PA	cp	Tf	x	
*Bryum radiculosum* Brid.	Xerophytic	Oceanic-mediterranean	P	ce	Tf	x	
*Calliergonella cuspidata* (Hedw.) Loeske	Hygro-mesophytic	Temperate	P	pc	We		x
*Campylopus brevipilus* Bruch & Schimp.	Hygro-xerophytic	Oceanic	P	c	Tuft		x
*Campylopus atrovirens* De Not.	Hygro-mesophytic	Oceanic-mediterranean	P	ps	Tuft		x
*Campylopus pilifer* Brid.	Xerophytic	Oceanic-mediterranean	P	l	Tuft		x
*Campylopus introflexus* (Hedw.) Brid.	Hygro-xerophytic	Suboceanic	P	d	Tuft		x
*Cephaloziella rubella* (Nees) Warnst.	Meso-xerophytic	Oceanic	P	c	Ms		x
*Cheilothela chloropus* (Brid.) Broth.	Xerophytic	Oceanic-mediterranean	P	c	Tf	x	
*Dialytrichia mucronata* (Brid.) Broth.	Hygro-mesophytic	Submediterranean-suboceanic	P	c	Tuft	x	
*Dicranella howei* Renauld & Cardot	Xerophytic	Oceanic-mediterranean	A	c	Tf	x	
*Dicranella staphylina* H.Whitehouse	Mesophytic	Suboceanic	A	ce	Tf		x
*Dicranella cerviculata* (Hedw.) Schimp.	Hygro-mesophytic	Boreal	P	c	Tf		x
*Dicranodontium asperulum* (Mitt.) Broth.	Hygrophytic	Subartic-subalpine	P	p	Tuft		x
*Dicranum scoparium* Hedw.	Hygro-mesophytic	Boreal	P	pc	Tuft		x
*Geheebia spadicea* (Mitt.) R.H.Zander	Hygrophytic	Temperate	P	c	Tf	x	
*Vinealobryum insulanum* (De Not.) R.H.Zander	Hygro-mesophytic	Submediterranean-suboceanic	PA	c	Tf	x	
*Geheebia lurida* (Hornsch.) J.A. Jiménez & M.J. Cano	Xerophytic	Submediterranean	P	c	Tf	x	x
*Entosthodon fascicularis* (Hedw.) Müll.Hal.	Hygro-mesophytic	Oceanic-mediterranean	A	a	Tf		x
*Ephemerum serratum* (Hedw.) Hampe	Hygro-mesophytic	Oceanic-mediterranean	A	a	Tp		x
*Ephemerum recurvifolium* (Dicks.) Boulay	Hygro-mesophytic	Submediterranean	A	a	Tp		x
*Ephemerum crassinervium* (Schwägr.) Hampe subsp. *sessile* (Bruch) Holyoak	Hygro-xerophytic	Suboceanic	A	a	Tp		x
Epipterygium *tozeri* (Grev.) Lindb.	Hygro-mesophytic	Oceanic-mediterranean	P	c	Ts	x	
Eurhynchiastrum *pulchellum* (Hedw.) Ignatov & Huttunen	Meso-xerophytic	Boreal	P	ps	Mr	x	
Fissidens *curvatus* Hornsch.	Hygro-mesophytic	Oceanic-mediterranean	P	c	Ts		x
Fissidens *bryoides* Hedw. var. *bryoides*	Hygro-xerophytic	Temperate	P	c	Tf		x
*Fissidens taxifolius* Hedw.	Mesophytic	Temperate	P	c	Tf	x	
*Fissidens dubius* P.Beauv. var. *dubius*	Mesophytic	Temperate	P	p	Tf		x
*Fossombronia wondraczekii* (Corda) Dumort. ex Lindb.	Hygro-mesophytic	Temperate	AP	a	Sc		x
*Fossombronia caespitiformis* (Raddi) De Not. ex Rabenh. subsp. *Caespitiformis*	Meso-xerophytic	Oceanic-mediterranean	PA	a	Sc		x
*Fossombronia pusilla* (L.) Nees	Hygro-mesophytic	Oceanic-mediterranean	AP	a	Sc		x
*Frullania dilatata* (L.) Dumort. subsp. *dilatata*	hygro-xerophytic	Temperate	P	l	Ms	x	
*Funaria hygrometrica* Hedw.	Hygro-mesophytic	Temperate	AP	f	Tuft	x	x
*Gongylanthus ericetorum* (Raddi) Nees	Hygro-xerophytic	Oceanic-mediterranean	AP	l	Sc		x
*Grimmia lisae* De Not.	Hygro-mesophytic	Mediterranean	P	c	Tf	x	
*Grimmia pulvinata* (Hedw.) Sm.	Xerophytic	Submediterranean	P	c	Cu	x	
*Grimmia laevigata* (Brid.) Brid.	Xerophytic	Submediterranean-suboceanic	P	c	Cu	x	
*Grimmia trichophylla* Grev.	Hygro-mesophytic	Temperate	P	cp	Cu	x	
*Homalothecium sericeum* (Hedw.) Schimp.	Xerophytic	Temperate	P	p	Mr	x	
*Homalothecium aureum* (Spruce) H.Rob.	Xerophytic	Mediterranean	P	p	Mr	x	
*Hypnum jutlandicum Holmen* & E.Warncke	Hygro-mesophytic	Suboceanic	P	p	Ms		x
*Hypnum cupressiforme* Hedw. var. *cupressiforme*	Meso-xerophytic	Temperate	P	ps	Ms	x	x
*Hypnum cupressiforme* Hedw. var. *lacunosum* Brid.	Xerophytic	Temperate	P	ps	Mr		x
*Hypnum resupinatum* Taylor	Mesophytic	Temperate	P	ps	Mr	x	
*Imbribryum alpinum* (Huds. ex With.) N.Pedersen	Hygro-mesophytic	Oceanic-mediterranean	P	c	Tf	x	x
*Isothecium myosuroides* Brid.	Hygro-mesophytic	Oceanic-mediterranean	P	ps	De	x	
*Kindbergia praelonga* (Hedw.) Ochyra	Hygrophytic	Temperate	P	p	Mr	x	
*Leptodictyum riparium* (Hedw.) Warnst.	Hygrophytic	Temperate	P	p	Mr	x	
*Nogopterium gracile* (Hedw.) Crosby & W.R.Buck	Hygro-xerophytic	Oceanic-mediterranean	P	l	Mr	x	
*Orthotrichum tenellum* Bruch ex Brid.	Xerophytic	Oceanic-mediterranean	P	c	Cu	x	
*Oxyrrhynchium speciosum* (Brid.) Warnst.	Hygro-mesophytic	Temperate	P	s	Mr	x	
*Phaeoceros laevis* (L.) Prosk.	Hygro-mesophytic	Oceanic-mediterranean	AP	a	Mt		x
*Physcomitrium pyriforme* (Hedw.) Bruch & Schimp.	Hygrophytic	Temperate	A	a	Tuft		x
*Plagiomnium ellipticum* (Brid.) T.J.Kop.	Hygrophytic	Boreal	P	pc	Tf		x
*Pleuridium acuminatum* Lindb.	Hygro-mesophytic	Suboceanic	PA	a	Tf		x
*Pohlia nutans* (Hedw.) Lindb. subsp. *nutans*	Hygro-mesophytic	Boreal	P	pc	Tuft		x
*Polytrichum formosum* Hedw.	Mesophytic	Temperate	P	pc	Tf		x
*Polytrichum juniperinum* Hedw.	Xerophytic	Temperate	P	pc	Tf		x
*Pseudocrossidium hornschuchianum* (Schultz) R.H.Zander	Meso-xerophytic	Submediterranean-suboceanic	P	c	Tf	x	
*Pseudoscleropodium purum* (Hedw.) M.Fleisch.	Mesophytic	Temperate	P	p	We		x
*Ptychostomum pseudotriquetrum* (Hedw.) J.R.Spence & H.P.Ramsay var. *pseudotriquetrum*	Hygrophytic	Temperate	P	pc	Tf	x	x
*Ptychostomum pallens* (Sw. ex anon.) J.R.Spence	Hygrophytic	Temperate	P	s	Tf	x	
*Ptychostomum capillare* (Hedw.) Holyoak & N.Pedersen	Meso-xerophytic	Temperate	P	c	Tf	x	x
*Racomitrium ericoides* (Brid.) Brid.	Hygro-mesophytic	Oceanic	P	cp	Tf		x
*Rhynchostegiella curviseta* (Brid.) Lindb.	Hygro-xerophytic	Submediterranean-suboceanic	P	ps	Ms	x	
*Rhynchostegium riparioides* (Hedw.) Cardot	Hygrophytic	Temperate	P	p	Ms	x	
*Rhynchostegium megapolitanum* (Blandow ex F.Weber & D.Mohr) Schimp.	Hygro-xerophytic	Submediterranean	P	p	Mr	x	
*Rhynchostegium confertum* (Dicks.) Schimp.	Hygro-mesophytic	Submediterranean-suboceanic	P	p	Mr	x	
*Riccardia chamedryfolia* (With.) Grolle	Hygrophytic	Oceanic	P	c	Mt		x
*Riccia canaliculata* Hoffm.	Hygro-mesophytic	Temperate	A	a	St	x	x
*Riccia crozalsii* Levier	Hygro-xerophytic	Oceanic-mediterranean	AP	a	St		x
*Riccia subbifurca* Warnst. ex Croz.	Meso-xerophytic	Submediterranean-suboceanic	AP	a	St		x
*Riccia sorocarpa* Bisch. subsp. *sorocarpa*	Meso-xerophytic	Temperate	AP	a	St	x	x
*Riccia beyrichiana* Hampe	Hygro-mesophytic	Oceanic-mediterranean	P	a	St		x
*Riccia bifurca* Hoffm.	Hygro-xerophytic	Submediterranean	P	a	St	x	
*Riccia nigrella* DC.	Hygro-xerophytic	Oceanic-mediterranean	PA	a	St	x	
*Riccia michelii* Raddi	Meso-xerophytic	Mediterranean	AP	a	St	x	
*Riccia gougetiana* Durieu & Mont. var. *gougetiana*	Hygro-xerophytic	Submediterranean	Ap	a	St		x
*Scleropodium cespitans* (Wilson ex Müll.Hal.) L.F.Koch	Sesonally hygrophytic	Oceanic-mediterranean	P	p	Ms	x	
*Scleropodium touretii* (Brid.) L.F.Koch	Xerophytic	Oceanic-mediterranean	P	p	Mr	x	
*Syntrichia princeps* (De Not.) Mitt.	Meso-xerophytic	Oceanic-mediterranean	P	c	Tf	x	
*Syntrichia laevipila* Brid.	Xerophytic	Oceanic-mediterranean	P	c	Tf	x	
*Tortella tortuosa* (Hedw.) Limpr.	Meso-xerophytic	Boreal	P	ps	Tuft		x
*Tortella squarrosa* (Brid.) Limpr.	Xerophytic	Submediterranean	P	cp	Tuft	x	x
*Tortula subulata* Hedw.	Meso-xerophytic	Boreal	P	c	Tuft		x
*Tortula truncata* (Hedw.) Mitt.	Meso-xerophytic	Temperate	AP	a	Tf	x	x
*Tortula muralis* Hedw. subsp. *muralis* var. *muralis*	Meso-xerophytic	Temperate	P	ce	Tf	x	x
*Tortula caucasica* Broth.	Mesophytic	Temperate	PA	a	Tf	x	
*Trichostomum brachydontium* Bruch	Meso-xerophytic	Submediterranean	P	p	Tf	x	

## 3. Materials and Methods

### 3.1. Study Sites

A comparative analysis of the bryophyte flora in wetlands of central Sardinia (*Pauli* of the Giara) and central Italy (Piana di Ferretto) was conducted (Figure 1). The *Pauli* of the Giara are small endoreic pools situated on a basalt plateau, whereas Piana di Ferretto is a plain region comprising small ponds and waterlogged soils on Plio–Pleistocene sandy–clayey sediments. These have resulted in the formation of oligotrophic, leached, and decarbonated soils, which are distributed across a mosaic of forests, heaths, agricultural fields, and small settlements. From a bioclimatic and biogeographic standpoint, Sardinia, located in a central position of the western Mediterranean area, is included in the Mediterranean bioclimatic region [13,19,20], western Mediterranean subregion, and Corsican–Sardinian province. The *pauli* site can be included in the meso-Mediterranean thermotype [21]. Ferretto is located in the submediterranean variant of the temperate bioclimatic region, meso-temperate thermotype [11,13]. Two hydrologic periods alternate in these temporary wetlands: one of flooding due to rainfall from winter to the end of spring and the other of drought owing to severe evaporation and dispersion of water during the summer and autumn. The vascular vegetation in these pools, which is strongly influenced by seasonal changes in the water level, is mainly represented by the classes *Isoëto-Nanojuncetea* and, to a lesser extent (especially in the inland sites), by fragments of *Littorelletea* [22]. Prior research has indicated that bryophytes exhibit a wide range of phenological patterns across various biogeographic regions, in contrast to vascular plants. Some bryophytes exhibit an early life cycle, resulting in their disappearance before the optimal period for the vascular taxa to develop fully. Conversely, other bryophytes emerge later in the season and persist until late spring [23]. Both sites have been extensively studied, and much bibliographic material is available, specifically for Ferretto floristic and vegetation data [11,23,24,25,26,27,28] and for Giara floristic and ecological data [21,29,30].

### 3.2. Type of Ponds

**Figure 4:**
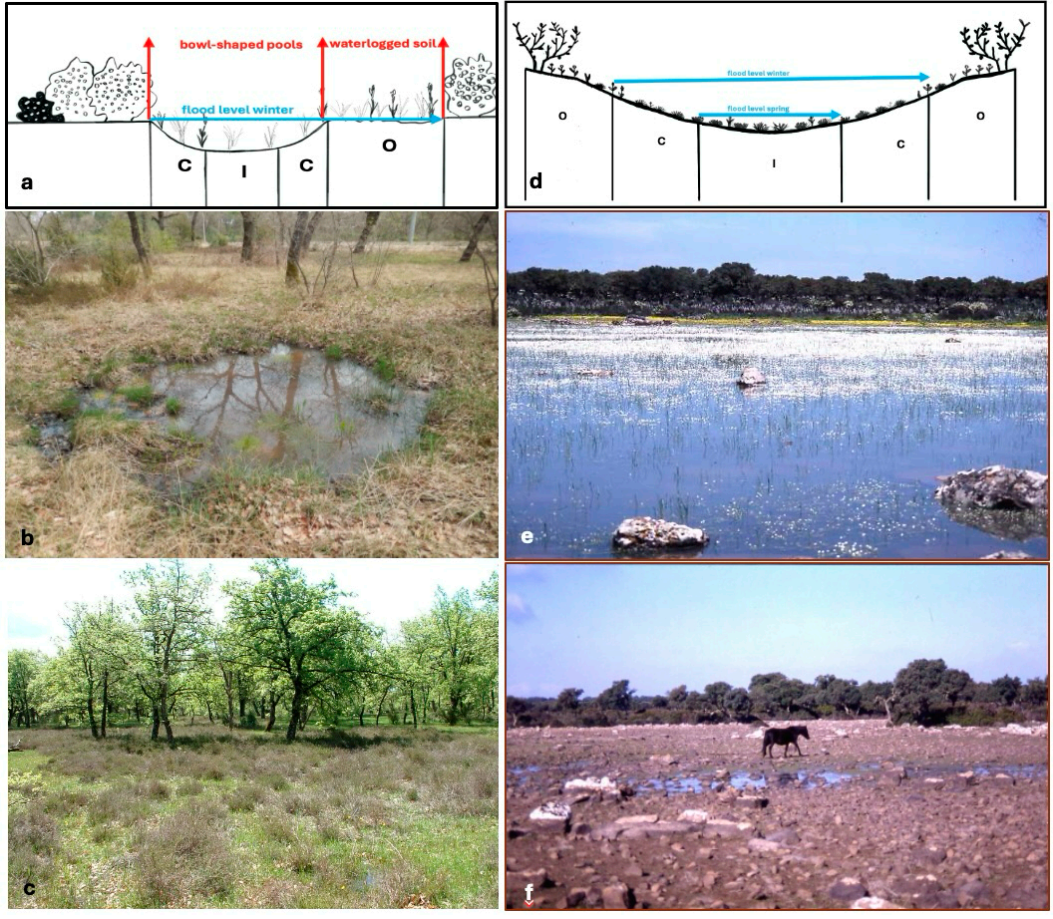
Type of ponds. (**a**) Graphical scheme of Ferretto-type pond: bowl-shaped pool and waterlogged soil with moisture belt distribution (I = inner, C = centre, and O = outer) drawn by S. Poponessi. (**b**) bowl-shaped pool; (**c**) waterlogged soil; (**d**) Graphical scheme of Giara-type pond with wide perimeter (water depth varies seasonally from 30 to 70 cm) and moisture belt distribution (I = inner, C = centre, and O = outer; modified [31]); (**e**) photo representing the winter flood level, and (**f**) photo representing the spring flood level.

Because the pools are located on different substrates in different biogeographical areas, their physiognomy is also different. Ferretto is a system of small bowl-shaped pools and waterlogged soils, scattered in a predominantly agricultural context with small forest and shrub patches. The pools have an average diameter of about 1 m (these are the inner and central belts) and varying depths from 2 to 40 cm, while the waterlogged soils have larger surfaces, of the order of a few to a few dozen square meters (this is the outer belt) [11]. *Pauli* is a system of pools and waterlogged soil on a basalt plateau. The pools are larger than those at Ferretto, and the water is about 30–70 cm deep [21]. The maximum water level varies depending on the size of the pool, and water stagnation lasts longer in larger pools. A comparison of the two types of pools is shown in Figure 4.

#### 3.2.1. Giara of Gesturi

The Giara of Gesturi is a site of the Natura 2000 Network (SAC ITB041112) and has an average altitude of 550 m a.s.l. (latitude 39°43′50″ and 39°47′52″ N, longitude 8°52′56″ and 9°01′00″ E). In the local vernacular language, the temporary pools are called “paule” or “piscina”. As testified by the widespread use of these names, temporary pools occupied larger areas in the past, but during the last centuries, they were reclaimed for agricultural activities [3,32].

MTPs are primarily situated within the expansive tablelands that bisect the topographical features and in the lowlands, where they are found within depressions that are characterised by clayey soil textures and slow drainage patterns across a broad altitudinal range (10–1100 m a.s.l.). 

The tableland landscape is typified by the prevalence of cork oak (*Quercus suber* L.) woods that are characterised by a neutral to acidic pH. These areas have been subjected to pastoral use and frequently transformed into wooded pastures, a process that has given rise to the dehesa landscape. Furthermore, the region is distinguished by the prevalence of deciduous oak woods, which are predominantly characterised by the *Quercus ichnusae* Mossa, Bacchetta, and Brullo. These organisms are particularly prevalent in central and northern Sardinia, occurring on non-carbonate substrates. 

The edaphic–mesophilous *Quercus ilex* L. is a dominant species in the lowland landscape, frequently occurring in association with the cork oak (*Q. suber*). These two species are particularly prevalent in floodplains with a mixed clay–sand matrix and moderately hydromorphic soils [33].

#### 3.2.2. Piana di Ferretto

The Piana di Ferretto is a vast, flat expanse situated between 260 and 320 metres above sea level on the west side of Lake Trasimeno, the largest lake in Italy. It is located in the Umbria region, in the vicinity of the city of Perugia. The site is of considerable conservation value, encompassing flora and fauna, as well as plant communities [11]. In consequence of the presence of a number of habitats included in Annex I of the 92/43/EEC Directive (in addition to the aforementioned 3170*, there are also 4030, 6420, 91M0, and 92A0), the site has been designated as a special area of conservation (SAC) belonging to the Natura 2000 network (code IT5210020). The underlying geological substrate can be identified as sandy–clayey Plio–Pleistocene sediments, which have their origins in oligotrophic, leached, and decarbonated soils [34]. During the rainy season, the argillaceous fraction of the soils retains water, which can result in flooding conditions. The final configuration of the pond system encompasses an area of approximately 2500 hectares and is situated within a complex mosaic of forests, heaths, agricultural fields, and small settlements. The water supply of the ponds is solely dependent on precipitation, which results in them becoming completely desiccated during the summer drought period.

### 3.3. Materials and Data Methods

The methodology employed for the bryophyte sampling was that which had previously been utilised for the vascular flora surveys of MTPs [30]. In order to ascertain the presence of bryophytes in the ponds, a programme of repeated visits was undertaken on a yearly and monthly basis (from October 2008 to October 2014 in Sardinia; from spring 2015 to spring 2016 at Piana di Ferretto) and we drew up a list of all the taxa present and where they occurred compared to the substrate moisture. In order to examine the spatial zonation of bryophytes within temporary ponds at the small-scale, we employed a methodology analogous to that previously used in Iberian and Moroccan ponds [5,31] and also used it for the vascular species in Sardinian ponds [3,29,35]. The method is based on three key factors: the depth of the water at the start of the dry season, the morphology of the pond, and the type of vegetation present. We investigated the distribution of bryophytes along concentric zones according to their humidity level. It was possible to identify an exceptionally flooded external zone (O—“Outer belt”), an intermediate zone (C—“Central belt”), and an internal zone (I—“Inner belt”) in which the flooding phase is prolonged. Where present, the waterlogged soil was treated as an outer belt. For each *taxon,* the Chorological element was considered based on the nomenclature established by [36]. The various elements were brought together considering the similarities in 12 major groups (arctic–alpine; subarctic–subalpine; suboceanic; boreal; oceanic; oceanic–mediterranean; mediterranean; submediterranean–suboceanic; submediterranean; temperate; continental; and subtropical) according to [18] who adapted the elements to the Mediterranean region. As shown in Table 1, the life cycle was considered using the nomenclature established by [15]. The life strategies were considered as well, using the nomenclature established by [16] as follows: fuggitive (f); annual shuttle (a); colonist (c); ephemeral colonist (ce); pioneer colonist (cp); short-lived shuttle (s); perennials (p); competitive perennials (pc); stress-tolerant perennials (ps); long-lived shuttle (l); dominant (d). The humidity needs for each taxon were taken from [11]. The life macroforms are in accordance with the classification by [15] which were grouped by the authors into seven macroforms, adapting the elements to the Mediterranean region, as follows: Cushion (dome-shaped colonies); Weft (intertwining branched layers); Dendroid (with stolons and erect shoots); Mat (creeping); Solitary (creeping solitary shoots and rosette-forming patch not mat); Turf (characterised by vertical stems with minimal branching; persistent protonema; displays a scattered vertical growth pattern); and Tuft (characterised by loose cushions and not shaped like a dome) (Figure 3). The classification used for each type of environmental parameter considered is shown in Table 1. The nomenclature of the taxa follows [37].

## 4. Conclusions

These habitats contribute to our knowledge of Annex I priority habitat 3170*, which includes a bryophyte component that has been often overlooked, although it is an emblematic feature of the habitat type and a crucial indicator, particularly for monitoring and management. MTP habitats are widespread in the coastal, subcoastal, and sometimes inland areas of peninsular and island Italy. They are also present in other regions of southern Europe, such as Greece, Spain, and Portugal. More generally, their distribution extends to central–western and south–eastern Europe and particularly in the arid and subarid areas of the Mediterranean basin [4]. In other European regions, such as northern Europe, similar temporary pond habitats exist, but with a more limited distribution [32].

In particular, these sites studied represent two emblematic examples that well embody the expression of this habitat in two different macroclimates; therefore, their interpretation can have a wide-ranging value, applicable to the whole Mediterranean territory and also to the transition areas between the Mediterranean and temperate macroclimate that occur in the entire Mediterranean basin. Water availability plays a fundamental role in MTP habitats, underpinning the fluctuation of the highly hygrophilous taxa in particular periods of the annual cycle [23,26,28,38]. In these delicate habitats, the bryophyte component is typically comprised of ephemeral liverworts, which exhibit a markedly dependent life cycle in response to variations in humidity and water fluctuations in the soil, largely as a result of the pluviothermic regimen. The ecological characteristics of the habitat cause a selection that allows the establishment of a flora characterised by liverworts, with the predominance of species of the genera *Riccia* and *Fossombronia* and mosses such as *Archidium*, *Pleuridium*, *Bryum*, *Imbribryum*, and *Ptychostomum*. These mosses are well adapted to the fluctuating water levels of their habitats. The abundant presence of liverworts with undeveloped sporophytes and mosses with cleistocarpous capsules make this flora very peculiar. The belts, where the distribution of species is based on the moisture gradient, play a fundamental role in the distribution of taxa.

**Figure 5:**
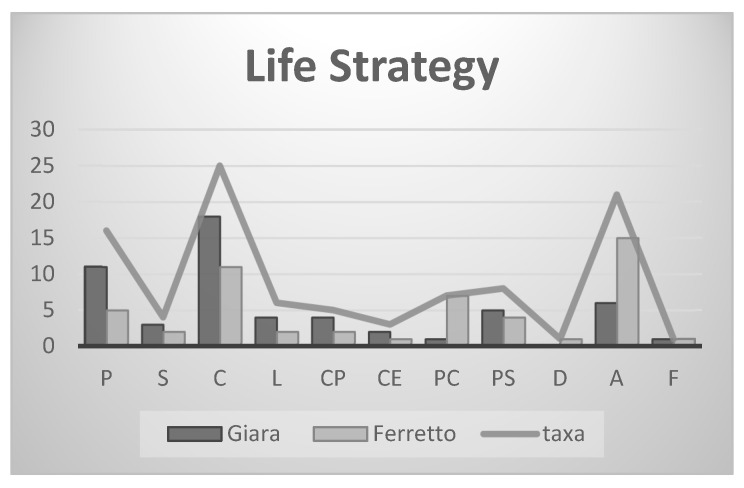
Relation of total number of taxa to life strategy for each site.

The majority of species observed at the Ferretto exhibit an “annual shuttle” life strategy, which is characterised by seasonal reproduction, large spores with limited dispersal capacity, and the absence of innovations (asexual propagation). This strategy is optimal for a habitat that is transient, yet predictable in its recurrence within the same location or in the surrounding area, maintaining a consistent community. The distribution of these species in the temporary ponds is particularly linked to the characteristics of the habitat. They prefer bare and pioneer environments but, above all, a well-humidified environment in winter and spring and one subjected to summer desiccation. At Giara, most species show a “colonist” life strategy, have a moderately short lifespan, and disappear into an unpredictable habitat (Figure 5). This result may be a consequence of the bioclimatic diversity between the sites, as well as of the size and depth of the pools, and of the type of substrate, behaving differently during seasonal water fluctuations. The pools of Giara are physiognomically different from those of Ferretto, especially in that they have a larger diameter and often greater depths. They are filled with rainwater in winter, which percolates through the basaltic cracks and then finds an outlet in the perimeter along the slope of the plateau, forming small waterfalls (called “mitzas”). Another portion of water evaporates, so that the spring–summer season creates the situation of waterlogged soil in the internal belt, and niches are observed as a result of the trampling and movement on the soil by grazing animals (Figure 3). Bryophytes have evolved morphological, physiological, and reproductive adaptations that enable them to thrive in wetland environments with varying hydroperiods. The frequency and magnitude of flooding events, along with water-level fluctuations, exert a significant influence on bryophyte species composition and life strategies. The distribution of species across the hydroperiod belts reflects their tolerance to flooding. The majority of species are found in the outer belt, which is less influenced by hydroperiods and water-level fluctuations. Consequently, it is the most stable and species-rich belt. A number of species belonging to this belt, including *Hypnum cupressiforme* var. *cupressiforme* Hedw., can be observed in the surrounding environments. The surrounding species are able to establish themselves in the outer belt, exploiting the low frequency of flooding to their advantage. The central and inner belts host a smaller number of species, and their presence is contingent upon fluctuations in water levels. Life strategies are employed to categorise bryophytes in accordance with their capacity to occupy, reproduce, and persist in a specific environment. Given the ephemeral nature of Mediterranean temporary habitats, life strategies inherently entail a series of trade-offs pertaining to a range of characteristics, including the capacity to withstand stressful environments, the preference for sexual or asexual reproduction, the length of the life cycle, and the size of the spore. It is noteworthy that there is a discernible gradient from the outer belt (O), which is predominantly composed of colonists and perennials, such as *Pottiaceae* and *Brachytheciaceae*, to the inner one (I), where annual shuttle species such as *Ricciaceae* are more prevalent. The floristic composition of the observed bryo-communities at Piana di Ferretto is impoverished when compared to occurrences of the same syntaxa in Mediterranean and subcoastal areas. This is due to the fact that the study area is located in a transitional territory from the climatic point of view, with Mediterranean traits of its climate resulting in a rather smoothed flora. This phenomenon, which is due to ecological and biogeographic reasons, is well known also for the vascular vegetation types colonizing the same habitat. This disturbance, at a low level of intensity, seems to positively affect the bryophytic component of the pools; indeed, the action of animals, causing an alteration in substrate morphology and the dynamism of the vascular vegetation, favours the settlement of entities of the genus *Riccia*. The bryophyte component that is most commonly found in these fragile habitats is primarily represented by ephemeral liverworts. These organisms have a life cycle that is severely dependent on fluctuations in humidity and water levels in the soil, which are largely influenced by the pluviothermic regime. The high vulnerability of these bryo-communities to climate change is a direct consequence of their strict dependence on climatic conditions. Our research findings indicate that, irrespective of the biogeographic context, the bryophytic taxa exhibit a highly distinctive phenology.

Our observations show that most of them have an early life cycle, with a tendency to disappear before the optimal developmental period for vascular taxa, while others appear later in time and persist until late spring. The outcomes strongly suggest that bryophytes need a tailored monitoring approach and special care in addressing nature conservation decisions, in order to reach substantial and satisfactory conservation targets. The main threats to bryophytes in MTPs appear to be a combination of anthropogenic pressures, land-use changes, lack of conservation efforts, and potential impacts of climate change. The diversity of bryophytes found in these temporary ponds is an important indicator of their conservation value. Furthermore, the presence of belt-specific indicator taxa and dominant species in distributed life forms/strategies in the various belts may be employed as effective proxies to monitor changes in flood levels over time.

## Data Availability

Data are contained within the article.

## References

[1] Williams P., Whitfield M., Biggs J., Bray S., Fox G., Nicolet P., Sear D. (2004). Comparative biodiversity of rivers, streams, ditches and ponds in an agricultural landscape in Southern England. Biol. Conserv..

[2] Deil U. (2005). A review on habitats, plant traits and vegetation of ephemeral wetlands—A global perspective. Phytocoenologia.

[3] Bagella S., Caria M.C. (2012). Diversity and ecological characteristics of vascular flora in Mediterranean temporary pools. Compt. Rend. Biol..

[4] Grillas P., Gauthier P., Yavercovski N., Perennou C. (2004). Mediterranean Temporary Pools: Issues Relating to Conservation, Functioning and Management.

[5] Rhazi L., Rhazi M., Grillas P., El Khyari D. (2006). Richness and structure of plant communities in temporary pools from western Morocco: Influence of human activities. Hydrobiologia.

[6] Blaustein L., Schwartz S.S. (2001). Why study ecology in temporary pools?. J. Zool..

[7] De Meester L., Declerck S., Stoks R., Louette G., Van De Meutter F., De Bie T., Michels E., Brendonck L. (2006). Ponds and pools as model systems in conservation biology, ecology and evolutionary biology. Aquat Concerv..

[8] Hulsmans A., Vanschoenwinkel B., Pyke C., Riddoch B.J., Brendonck L. (2008). Quantifying the hydroregime of a temporary pool habitat: A modelling approach for ephemeral rock pools in SE Botswana. Ecosystems.

[9] Article 17 Web Tool, 2024. Habitat Assessments at Member State Level. https://nature-art17.eionet.europa.eu/article17/habitat/report/.

[10] Janssen J.A.M., Rodwell J.S., Criado M.G., Gubbay S., Haynes T., Nieto A., Sanders N., Landucci F., Loidi J., Ssymank A. (2016). European Red List of Habitats. Part 2. Terrestrial and Freshwater Habitats.

[11] Gigante D., Maneli F., Venanzoni R. (2013). Mediterranean temporary wet systems in inland Central Italy: Ecological and phytosociological features. Plant Sociol..

[12] Poponessi S., Della Bella V. (2019). BOX 4. Stato delle conoscenze sulle briofite aliene invasive in Umbria. Caratterizzazione e Diffusione Delle Specie aliene Acquatiche e di Ambienti Umidi in Umbria.

[13] Pesaresi S., Biondi E., Casavecchia S. (2017). Bioclimates of Italy. J. Maps.

[14] Cogoni A., Filippino G., Marignani M. (2016). Small-scale pattern of bryoflora in Mediterranean temporary ponds: Hints for monitoring. Hydrobiologia.

[15] Hill M.O., Preston C.D., Bosanquet S.D.S., Roy D.B. (2007). BRYOATT: Attributes of British and Irish Mosses, Liverworts and Hornworts.

[16] Dierßen K. (2001). Distribution ecological amplitude and phytosociological characterization of European bryophytes. Bryophyt. Biblioth..

[17] Sérgio C., Casas C., Brugués M. (1994). Red List of Bryophytes of the Iberian Peninsula.

[18] Sérgio C., Garcia C.A., Vieira C., Hespanhol H., Sim-Sim M., Stow S., Figueira R. (2014). (Conservation of Portuguese red-listed bryophytes species in Portugal: Promoting a shift in perspective on climate changes. Plant Biosyst. Int. J. Deal. All Asp. Plant Biol..

[19] Pesaresi S., Galdenzi D., Biondi E., Casavecchia S. (2014). Bioclimate of Italy: Application of the worldwide bioclimatic classification system. J. Maps.

[20] Rivas-Martínez S., Fernández-González F., Loidi J., Lousã M., Penas A. (2001). Syntaxonomical chec- klist of vascular plant communities of Spain and Portugal to association level. Itin. Geobot..

[21] Cogoni A., Flore F., Adamo C., Lai R., Scrugli A. (2006). Ecology of bryophytes of damp areas at “Giara di Gesturi” (Southern Central Sardinia). Bocconea..

[22] Lorenzoni C., Paradis G. (1997). Phytosociologie de Mares Temporaires Méditerranéennes: Les Tre Padule et la Padule Maggiore (Suartone, commune de Bonifacio, Corse). Coll Phytosoc..

[23] Poponessi S., Aleffi M., Gigante D., Venanzoni R. (2016). Updates on the bryophyte flora of the lowland woods and temporary ponds west of Lake Trasimeno (Central Italy). Fl. Medit..

[24] Aleffi M. (1992). Florula briologica dei boschi planiziari acidofili a sud del Lago Trasimeno (Umbria). Arch. Bot. Ital..

[25] Cortini Pedrotti C. (1985). La florule bryologique des collines sablonneuses a l’ouest du lac Trasimene (Ombrie). Cryptog. Bryol. Lichénol..

[26] Ellis L.T., Agcagil E., Kırmacı M., Aleffi M., Bakalin V.A., Bednarek-Ochyra H., Cykowska-Marzencka B., Stryjak-Bogacka M., Bojaca G.F.P., Fantacelle L.B. (2016). New National and Regional Bryophyte Records, 49. J. Bryol..

[27] Pedrotti F. (1982). La vegetation des collines entre le Trasimene et le Val de Chiana. Guide–Itinéraire, Excursion Internatio- nale de Phytosociologie en Italie centrale, Pedrotti, F. (2–11 juillet 1982).

[28] Poponessi S., Aleffi M., Maneli F., Venanzoni R., Gigante D. (2018). Bryophytic vegetation of fragile and threatened ecosystems: The case of the Mediterranean temporary ponds in inland Central Italy. Plant Sociol..

[29] Bagella S., Caria M.C., Farris E., Filigheddu R. (2009). Phytosociological analysis in Sardinian Mediterranean temporary wet habitats. Fitosociologia.

[30] Bagella S., Caria M.C., Farris E., Filigheddu R. (2009). Spatial-time variability and conservation relevance of plant communities in Mediterranean temporary wet habitats: A case study in Sardinia (Italy). Plant Biosyst..

[31] Casas C., Cros R.M., Brugue´s M., Sergio C., Font J. (1998). Els briofits de les basses de l’Albera, Alt Emporda. Gea Flora Et Fauna.

[32] Bagella S., Caria M.C. (2013). Sensitivity of ephemeral wetland swards with Isoetes histrix Bory to environmental variables: Implications for the conservation of Mediterranean temporary ponds. Aquat. Conserv. Mar. Freshw. Ecosyst..

[33] Bacchetta G., Bagella S., Biondi E., Farris E., Filigheddu R., Mossa L. (2009). Vegetazione forestale e serie di vegetazione della Sardegna (con rappresentazione cartografica alla scala 1:350,000). Fitosociologia.

[34] Giovagnotti C., Calandra R., Leccese A., Giovagnotti E. (2003). I Paesaggi Pedologici e la Carta dei Suoli dell’Umbri.

[35] Bagella S., Caria M.C., Zuccarello V. (2010). Patterns of emblematic habitat types in Mediterranean temporary wet- lands. Compt. Rend. Biol..

[36] Düll R. (1983). Distribution of European and Macaronesian mosses (Bryophytina). Bryologische Beiträge.

[37] Aleffi M., Cogoni A., Poponessi S. (2023). An updated checklist of the bryophytes of Italy, including the Republic of San Marino and Vatican City State. Plant Biosyst. Int. J. Deal. All Asp. Plant Biol..

[38] Ernandes P., Marchiori S. (2013). Mediterranean tem-porary ponds in Puglia (South Italy): A “joyau flo-ristique” to protect. Acta Bot. Gall..

